# Molecular characterization of methicillin-resistant *Staphylococcus aureus* genotype ST764-SCC*mec* type II in Thailand

**DOI:** 10.1038/s41598-022-05898-1

**Published:** 2022-02-08

**Authors:** Sumalee Kondo, Pimonwan Phokhaphan, Sissades Tongsima, Chumpol Ngamphiw, Worawich Phornsiricharoenphant, Wuthiwat Ruangchai, Areeya Disratthakit, Pholawat Tingpej, Surakameth Mahasirimongkol, Aroonlug Lulitanond, Anucha Apisarnthanarak, Prasit Palittapongarnpim

**Affiliations:** 1 Faculty of Medicine, Thammasat University, Pathum Thani, 12120 Thailand; 2 National Biobank of Thailand (NBT), National Science and Technology Development Agency, Pathum Thani, 12120 Thailand; 3 Pornchai Matangkasombut Center for Microbial Genomics, Mahidol University, Bangkok, 10400 Thailand; 4 Medical Life Science Institute, Ministry of Public Health, Nonthaburi, 11000 Thailand; 5 Faculty of Associated Medical Sciences, Khon Kaen University, Khon Kaen, 40002 Thailand

**Keywords:** Microbiology, Molecular biology

## Abstract

Methicillin-resistant *Staphylococcus aureus* (MRSA) is a significant causative agent of hospital-acquired infections. We characterized MRSA isolated from August 2012 to July 2015 from Thammasat University Hospital. Genotypic characterization of MRSA SCC*mec* type II and III isolates were scrutinized by whole genome sequencing (WGS). The WGS data revealed that the MRSA SCC*mec* type II isolates belonged to ST764 previously reported mainly in Japan. All of tested isolates contained ACME Type II′, SaPIn2, SaPIn3, *seb*, interrupted *SA1320*, and had a virulence gene profile similar to Japan MRSA ST764. Rigorous surveillance of MRSA strains is imperative in Thailand to arrest its potential spread.

## Introduction

Methicillin-resistant *Staphylococcus aureus* (MRSA) is a major cause of hospital-acquired infection (HA-MRSA) worldwide. There have been increasing reports of community-acquired MRSA (CA-MRSA)^1^ and livestock-associated MRSA (LA-MRSA)^2^. The resistance is generally conferred by the acquisition of a SCC*mec* element, which carries *mecA*, a gene encoding for PBP-2′, a penicillin-binding protein that is not inhibited by most beta-lactam antibiotics. The SCC*mec* element may also carry resistance genes to other classes of antibiotics such as aminoglycosides and glycopeptide antibiotics^3^. Many MRSA strains have become global multidrug resistant. For instance, MRSA strains isolated from a tertiary hospital in Malaysia showed co-resistance of tetracycline, ciprofloxacin and erythromycin^4^. MRSA-infected patients have a higher mortality rate than those infected with susceptible strains. Treatment of MRSA infection relies primarily on vancomycin. Although *S*. *aureus* isolates with complete resistance to vancomycin (VRSA) are still not common, milder vancomycin resistance phenotypes may cause problems in treatment. These include vancomycin-intermediate resistant *S*. *aureus* (VISA), heterogeneous vancomycin-intermediate resistant *S*. *aureus* (hVISA), and even the strains with reduced susceptibility to vancomycin at the MIC level of 1.5–2.0 µg/ml (vancomycin MIC creep)^5–7^. Strains with decreased susceptibility to vancomycin were reported to be a cause of treatment failure^8^. We selected the MRSA isolates by various methods. Despite the MRSA ST239 SCC*mec* type III genotype being commonly reported in Thailand, 41% of MRSA in Thammasat University Hospital (TUH)^9^ was SCC*mec* type II MRSA (unpublished data), which differed from other tertiary hospitals in Thailand^10,11^.

In this study, five isolates of HA-MRSA SCC*mec* type II and two isolates of SCC*mec* type III were whole-genome sequenced. All of the MRSA SCC*mec* type II isolates belonged to an unexpected MLST type, ST764 which had been reported mostly in Japan. The MRSA ST764 strains frequently contained ACME type II^12^. This strain was first discovered in a community setting in Japan in 2009 as a variant of ST5 genotype with SCC*mec* type II/*spa*2/*seb*2/PVL-^13^. The ST764 genotype was later reported as a common genotype in many settings in Japan, including tertiary hospitals^14,15^, outpatient departments^16^, long-term care facilities^17^ and communities^18^, and this genotype has been increasing^16^.

This study reports, for the first time, WGS data of MRSA ST764 SCC*mec* type II in Thailand, using Illumina NextSeq 500 platform. All isolates here were from severe hospital-acquired infection patients with high mortality. Although these MRSA strains were still susceptible to vancomycin, their MICs of vancomycin were mostly at the high end of the susceptible range. The discovery in Thailand of the ST764 genotype, a well-known Japan CA-MRSA, indicates the increase in the geographic range of the bacteria beyond East Asia. Since the ST764 genotype can be community-acquired, this emerging clone poses a significant threat not only to TUH but also to the other hospitals in the region. The presence of this MRSA strain warrants vigorous surveillance in hospitals and immediate investigation of community transmissions of the strains.

## Results

Five isolates of HA-MRSA SCC*mec* type II [SATU130 (SRA: SRR16971216), SATU131 (SRA: SRR16971215), SATU132 (SRA: SRR16971214), SATU134 (SRA: SRR16971213) and SATU136 (SRA: SRR16971212)] and two isolates of HA-MRSA SCC*mec* type III [SATU133 (SRA: SRR17033804) and SATU135 (SRA: SRR17033804) were whole-genome sequenced. All isolates were from patients with invasive diseases, including pneumonia, septicemia, osteomyelitis and endocarditis as described in Table 1. The majority of the patients were over 60 years old. All five SCC*mec* type II isolates belonged to the ST764 genotype while the SCC*mec* type III isolates belonged to ST22 and ST239. None of the patients was HIV seropositive, but four patients with ST764 infection had diabetes with chronic kidney diseases. Only one patient with MRSA ST764 infection survived.

**Table 1:** Profiles of patients whose MRSA isolates were studied by WGS.

Strain code	ST	Date of isolation	Age	Sex	Diagnosis	Sample type	Days to negative culture	Underlying diseases		Treatment outcomes
								DM	CKD	
SATU130	ST764	10/10/2013	84	M	Septicemia	Blood	8	Yes	Yes	Death
SATU131	ST764	5/11/2013	78	M	Pneumonia	Urine	8	No	No	Death
SATU132	ST764	9/4/2013	61	M	Pneumonia	Sputum	19	Yes	Yes	Success
SATU133	ST239	1/2/2013	18	M	Osteomyelitis of left femur	Bone	17	No	No	Success
SATU134	ST764	15/1/2013	80	F	Pneumonia	Sputum	13	Yes	Yes	Death
SATU135	ST22	21/2/2015	78	M	Pneumonia	Sputum	NA	No	No	Success
SATU136	ST764	29/1/2014	80	F	Endocarditis and septicemia	Blood	8	Yes	Yes	Death

*WGS* whole genome sequencing, *ST* sequence type, *NA* not available, *DM* diabetes mellitus, *CKD* chronic kidney disease.

All isolates were resistant to cefpirome and clindamycin yet were susceptible to vancomycin, trimethoprim-sulfamethoxazole, linezolid, mupirocin and teicoplanin. However, MICs of vancomycin against most ST764 isolates were relatively high at 1.5–2.0 μg/ml. The MICs of the drugs against each isolate were shown in Table 2.

**Table 2:** Minimal inhibitory concentrations of antibiotics against the MRSA isolates.

Strain code	VAN	CPR	CLI	TMP	SXT	RIF	MUP	FUS	TEC	LZD
SATU130	1.5	> 256	> 256	0.5	0.012	0.012	0.094	0.125	0.75	2
SATU131	2	> 256	> 256	0.38	0.012	0.004	0.25	0.094	1	1
SATU132	0.75	> 256	> 256	0.25	0.008	0.008	0.094	0.25	1	1.5
SATU133	2	> 256	> 256	> 32	1	> 32	< 0.064	0.094	1	0.75
SATU134	1.5	> 256	> 256	0.75	0.016	0.008	< 0.064	0.19	0.38	1
SATU135	0.38	> 256	0.125	0.38	0.032	0.008	0.19	0.19	0.5	1.5
SATU136	2	> 256	> 256	0.38	0.012	0.008	0.19	0.125	0.5	1.5

*VAN* vancomycin, *CPR* cefpirome, *CLI* clindamycin, *TMP* trimethoprim, *SXT* trimethoprim-sulfamethoxazole, *RIF* rifampicin, *MUP* mupirocin, *FUS* fusidic acid, *TEC* teicoplanin, *LZD* linezolid.

The isolates were characterized genetically by SCC*mec* typing, *spa* typing, MLST, and WGS with the results shown in Supplementary Table S1. Isolates belonging to ST239 and ST22 harbored SCC*mec* type III while all ST764 isolates harbored SCC*mec* type II, similar to previous reports^14,19,20^.

### Characterization of single nucleotide variants (SNVs)

More than 90% of reads were uniquely mapped to *S. aureus* N315 (ASM964v1, GenBank accession: BA000018.3), an ST5 isolate. The mean coverage of the sequence was 93% with 72X average depth. A total of 50,021 SNVs were identified based on alignment to the reference genome of *S. aureus* N315. The number of SNVs identified among ST764 isolates ranged from 237 to 261. The average pairwise SNV distance among ST764 isolates was 49.8.

**Figure 1:**
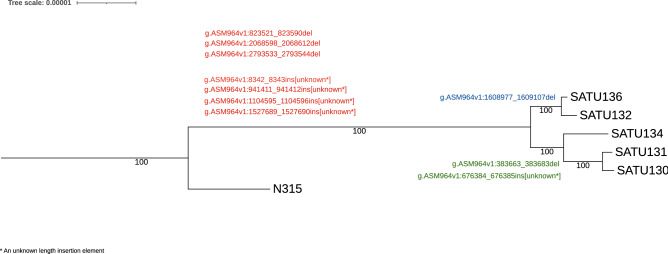
An ML phylogenetic tree of five ST764 isolates with N315 (ST5) as the outgroup. The deletions and insertions were labeled alongside with the branches shared by all isolates that shared the deletions/insertions as indicated with red (common to all isolates), blue and green. The HGVS nomenclature (available from http://varnomen.hgvs.org/) were used to describe the deletion and insertion events. The “unknown” indicated in the nomenclature means unknown inserted sequences.

The maximum likelihood phylogenetic tree of five ST764 isolates, with N315 as the outgroup, was shown in Fig. 1. There were two pairs, SATU130–SATU131 and SATU132–SATU136, with the same SNV distances of 11 (Table 3), suggesting epidemiological linkages between each pair^21^. SATU130 and SATU131 were isolated from the same male surgical ward, 26 days apart, and therefore probably belonged to the same clones. Isolates SATU132 and SATU136 were from male and female medicine wards, 9 months apart. Moreover, isolate SATU136 did not contain *seb*, while SATU132 did. The epidemiological link between the two isolates was therefore uncertain.

**Table 3:** Number of SNV differences between each pair of isolates.

	N315	SATU130	SATU131	SATU132	SATU134	SATU136	SATU133	SATU135
N315								
SATU130	261							
SATU131	258	11						
SATU132	238	67	66					
SATU134	258	49	48	64				
SATU136	237	62	61	11	59			
SATU133	22,720	22,893	22,890	22,880	22,897	22,877		
SATU135	31,919	32,087	32,084	32,074	32,093	32,071	35,871	

All five ST764 isolates shared three deletions (g.ASM964v1:823521_823580del, g.ASM964v1:2068598_2068612del, g.ASM964v1:2793533_2793544del) and four insertions (g.ASM964v1:8342_8343ins [unknown], g.ASM964v1:941411_941412ins [unknown], g.ASM964v1:1104595_1104596ins [unknown], g.ASM964v1:1527689_1527690ins [unknown]), which were not present in the ST22 and ST239 isolates. There were some indels found in some but not all ST764 isolates. A deletion (g.ASM964v1:1608977_1609107del) was shared by SATU132 and SATU136 while a deletion (g.ASM964v1:383663_383683del) and an insertion (g.ASM964v1:676384_676385ins [unknown]) could be found only among isolates SATU130, SATU131, and SATU134, as shown in Fig. 1 and Supplementary Tables S2 and S3.

### Characterization of SCC*mec*-ACME II′ composite island of ST764

We investigated the presence of various genes and genetic elements previously described in ACME**-**SCC*mec* composite island (ACME**-**SCC*mec* CI)^12^. All ST764 isolates here contained *arcA*–*D*, but not *opp3*; thus the ACME element was classified as ACME II′. They did not harbor any SNV in *arcA*–*D* as compared with N315, which was similar to those Japan ST764 isolates. It should be noted that C4913T mutation in *arcB* was found in an ST764 isolate in Japan^22^. ACME**-**SCC*mec* CI was previously classified into five types. The five ST764 isolates harbored cJR1 DNA segment but carried neither pUB110 nor *ccrC*. Thus, these isolates belonged to type A3^23^. The ACME-SCC*mec* CI type A3 is the shortest form of the complex and probably puts less burden on the fitness cost for the bacteria.

### The presence of genetic elements specific to ST764

In order to further evaluate similarity between the ST764 isolates in this study and the isolates reported from Japan, we identified other genetic elements previously described for ST764 in Japan apart from the ACME-SCC*mec* CI.

The five ST764 isolates contained SaPIn2 and SaPIn3 pathogenic islands. The former exotoxin island, SaPIn2, contained *set*6 to *set15*. The latter enterotoxin island contained *seg*, *sei, sem*, *sen*, and *seo*. These isolates did not have SAPIn1, evidenced by the absence of *tst*, *sea, sec*, *sel* or *sek*. Our ST764 isolates did not contain *sed, see, seq,* or *sep* either. They did not contain exfoliative toxin genes (*eta* and *etb*). These ST764 isolates were characterized as having *cna* with *pvl*-negative but also having *lukE*-luk*D***.** These isolates contained *hla*, *hld* and *hlgA*-*C*. Such genetic characteristics matched the genetic profile of the ST764 isolates in Japan. The Thai isolates contained adhesin genes, *icaA*, *sdrC***-***E*, *ebpS*, *clfA*, *clfB*,—*bbp*, *fnbA* and *fnbB* (partial sequence).

The ST764 isolates in Japan were described as containing three more insertions, SaPInn54-13.5 kb fragment containing *seb,* prophage carrying *SA1320* gene, and *tet*(M)-carrying Tn5251. All except one ST764 here harbored *seb*, which was reported missing in some ST764 isolates in Japan^22^. All ST764 isolates here harbored the insertion in *SA1320*, *tet*(M) and Tn5251. The similarity between genetic profiles of the isolates studied here and the MRSA ST764 SCC*mec* type II in Japan suggests that the isolates in Thailand were from the outbreak clone in Japan.

## Discussion

HA**-**MRSA is a global problem, especially in large tertiary hospitals. Previous WGS studies in Thailand focused mainly on ST239 SCC*mec* type III, which was the most common MLST type^24–26^. It is, therefore, intriguing to find that SCC*mec* type II MRSA was common in TUH, unlike in other tertiary hospitals in Thailand^10,11^. Moreover, all belonged to ST764-MRSA-II and were from patients with severe hospital**-**acquired invasive diseases. The apparently high incidence remains to be confirmed.

ST764-MRSA-II, is a recently recognized genotype, reported as a common MRSA genotype in many settings in Japan and might be spreading^16^. Such hospital settings encompass outpatient departments and healthcare professionals^27^. This spreading may be attributed to the presence of ACME type II, which may provide the bacteria a better ability to colonize on skin and mucous membrane which could consequently lead to better transmission. It has recently been reported as a common genotype in Shanghai^28^ and Eastern China^29^ but not yet in Southern China^30^.

ST764 is a hybrid variant of the globally disseminated ST5 lineage of HA-MRSA, carrying a few virulence traits similar to community-acquired MRSA (CA-MRSA). ST764 has evolved via multiple steps, including the acquisition of ACME *arcA* and the staphylococcal enterotoxin B gene (*seb*) in SaPInn54^12^.

Here we reported, for the first time, the WGS of the five MRSA SCC*mec* type II isolates identified from 2013 to 2015 and demonstrated that all ST764 isolates in this study carried ACME-SCC*mec* CI type A3 similar to some ST764 isolates in Japan^23^. The ST764 strains from Thailand and those from Japan also shared other genetic characteristics, such as the lack of *pvl, tst, sec, sel* and *sep* with the presence of SaPIn2, SaPIn3, *seb*, *tet*(M)^12^. This implies that the ST764-MRSA-II clone in Thailand was similar to and probably originated from the clone in Japan.

The genetic similarity of ST764-MRSA-II in Thailand to ST764-MRSA-II in Japan suggested a possibility that the ST764-MRSA-II strain was brought to Thailand by a healthy carrier, and later was transmitted into the hospital settings. There is also a possibility that the ST764-MRSA-II strain may continue to spread wider via colonization of healthy carriers.

Analysis of whole genome sequencing data suggested potential transmission between a pair of isolates. The isolates had a SNP-distance of 11 and were isolated from the same ward within a month. This finding of MRSA genotype ST764-SCC*mec* type II is the first HA occurrence reported in Southeast Asia. Two isolates of CA ST764-SCC*mec* type II from Thai pig farm workers were also reported^31^. The potential danger of spreading necessitates rigorous surveillance of emerging MRSA strains.

## Methods

### Bacterial strains

One-hundred and one clinical isolates of MRSA, confirmed by the detection of *mecA*, at TUH from August 2012 to July 2015^9^, were characterized for SCC*mec* types, pulsed-field gel electrophoresis (PFGE) patterns, virulence genes patterns, and antibiotic resistance phenotypes. There were 41 samples belonging to SCC*mec* type II that revealed either PFGE pattern A or P. Five isolates with both A- and P-PFGE patterns were randomly selected for sequencing, together with two isolates belonging to SCC*mec* type III. Bacterial collection, demographic data, clinical and microbiological data retrieval from Microbiology laboratory, Thammasat University Hospital (TUH) were carried out in accordance with relevant guidelines and regulations after approval of the Human Research Ethics Committee of Thammasat University (approval no. MTU-EC-DS-1-193/63 and MTU-EC-DS-4-197/63) and TUH.

### Microbiological and clinical data

Demographic data, clinical and microbiological data were retrieved from MRSA-infected patients’ record files from the TUH database. Examples of patients’ clinical data include lengths of stay, clinical outcomes and underlying diseases including diabetes mellitus (DM) and chronic kidney diseases (CKD). Successful treatment outcomes referred to events when the patients were cured or improved and MRSA was eradicated. Outcomes of persistent-MRSA, recurrent-MRSA or death due to MRSA were regarded as failure of treatments. The patients who were diagnosed with MRSA infection at an outpatient clinic or within 48 h of hospitalization without history of exposure to any healthcare facilities during the previous two months were defined as community-acquired MRSA infection (CA-MRSA). Hospital-acquired MRSA infection (HA-MRSA) referred to those patients who became infected after 48 h of hospitalization.

### Antimicrobial susceptibility testing

Minimum inhibitory concentration (MIC) of vancomycin (VAN) was carried out by both broth microdilution method^32,33^ and E-test for determining the high end MICs. Other antibiotics including cefpirome (CPR), clindamycin (CLI), trimethoprim (TMP), trimethoprim-sulfamethoxazole (SXT), rifampicin (RIF), mupirocin (MUP), fusidic acid (FUS), teicoplanin (TEC) and linezolid (LZD) were tested by E-test (Liofilchem^®^, Italy).

### PCR detection of *S. aureus mecA* and *pvl* genes

Total DNA was purified from each overnight *S. aureus* culture using Genomic DNA Extraction Mini Kit (RBC Bioscience, New Taipei City, Taiwan). Amplification of *mecA* (5′-TCC AGA TTA CAA CTT CAC CAG G-3′ and 5′-CCA CTT CAT ATC TTG TAA CG-3′)^34^ and *pvl* (5′-ATC ATT AGG TAA AAT GTC TGG ACA TGA TCC A-3′ and 5′-GCA TCA AST GTA TTG GAT AGC AAA AGC- 3′)^35^ (Integrated DNA Technologies, Singapore) was carried out using the following conditions. The PCR mixture consisted of 50 μl of 10X PCR buffer, 50 mM MgCl_2_, 10 mM dNTPs, 100 μM specific primer pair and 1.25 U *Taq* polymerase (RBC Bioscience). Thermo-cycling was conducted in a MyCycler Thermal Cycler (Bio-Rad, Hercules, CA) as follows: 94 °C for 2 min; followed by 30 cycles of 94 °C for 30 s, 51 °C (for *mecA*) or 56 °C (for *pvl*) for 30 s, and 72 °C for 1 min; with a final elongation step at 72 °C for 5 min. Amplicons (162 bp and 433 bp of *mecA* and *pvl,* respectively) were analyzed by 1% agarose gel electrophoresis, stained with GelStar™ Nucleic Acid Gel Stain (Lonza Rockland, Rockland, ME) and visualized under UV light. *S. aureus* N315 and KKU-MS14 strains were used as *mecA-* and *pvl*-positive control, respectively.

### SCC*mec* typing

SCC*mec* typing was carried out using a multiplex PCR assay. Specific primers for SCC*mec* types I, II, III, and IVc were included **(**Supplementary Table S4)^36^. Positive controls, SCC*mec* type I (613 bp); SCC*mec* type II (398 bp) and SCC*mec* type III (280 bp), were added.

### Whole-genome sequencing

DNA purity was assessed using a spectrophotometer, NanoDrop™ 2000 (Thermo Fisher, USA) and DNA concentration was determined by measuring fluorescence in a fluorometer, Qubit™ 3 Fluorometer (Invitrogen™, USA). DNA integrity was visualized by running the isolated samples in agarose gel electrophoresis to assess extent of DNA shearing.

A DNA input of 1 ng was used to prepare a 150-base read library, using the Nextera XT DNA Library Preparation Kit (Illumina, USA). The library preparation protocol includes tagmentation of genomic DNA, library amplification, library clean up, library normalization and library pooling. Library denaturation and dilution were performed, and then diluted PhiX control V3 was spiked into the libraries to obtain 1% PhiX as an external control. The prepared library pool was loaded into the reagent cartridge and then sequenced by 150 bp paired-end NextSeq 500 sequencing (Illumina, USA). Sequences of the five isolates of HA-MRSA SCC*mec* type II were submitted to SRA. The accession numbers of each isolate were listed as follows. SATU130 (SRA: SRR16971216), SATU131 (SRA: SRR16971215), SATU132 (SRA: SRR16971214), SATU134 (SRA: SRR16971213) and SATU136 (SRA: SRR16971212)] and two isolates of HA-MRSA SCC*mec* type III [SATU133 (SRA: SRR17033804) and SATU135 (SRA: SRR17033804).

### Bioinformatic analyses

The sequencing reads were quality filtered using Trimmomatic version 0.39^37^ with the following parameters: LEADING: 3 TRAILING: 3 SLIDINGWINDOW: 4:20 MINLEN: 36. The quality-passed reads were aligned to the MRSA ST5 N315 reference genome (ASM964v1, GenBank accession: BA000018.3) using Burrows-Wheeler Aligner (BWA) version 0.7.17^38^ with the BWA-MEM algorithm. Sequencing read duplication was handled by Picard Toolkit version 2.18.27 (available from http://broadinstitute.github.io/picard/). For each MRSA isolate, variant identification of single nucleotide variants (SNVs) and short insertion-deletions (Indels) was carried out using the HaploytypeCaller module of Genome Analysis Toolkit (GATK) version 4.1.4.1^39^. Low quality variants that have read depth lower than 10 were discarded. Variant annotation was performed using SnpEff version 4.3^40^. Structural variants (SVs) were identified by running the program Manta^41^ on an input BAM file (sorted and mapped reads) that has aligned to the reference sequence. Resulting SV events flagged with “IMPRECISE” were discarded. The remaining SV events were confirmed by visualizing them using integrative genomics viewer (IGV)^42^. De novo Genome assembly was done by SPAdes3.9 (available from http://cab.spbu.ru/software/spades/) (Supplementary Fig. S1).

### Multilocus sequencing typing (MLST)

MLST was done by identifying the presence of variants located in seven housekeeping genes, carbamate kinase (*arcC*), shikimate dehydrogenase (*aroE*), glycerol kinase (*glp*), guanylate kinase (*gmk*), phosphate acetyltransferase (*pta*), triosephosphate isomerase (*tpi*), and acetyl coenzyme A acetyltransferase (*yqiL*)^43^. Parts of the assembled contigs from each isolate were aligned with *Staphylococcus aureus* MLST primers (available from https://pubmlst.org/organisms/staphylococcus-aureus/primers). The allelic profiles of our isolates were matched with allele sequences present in the PubMLST database (available from https://pubmlst.org/)^44^ (Supplementary Fig. S1).

### *Spa* typing

The *spa* sequence types were assigned using spaTyper1.0 (available from https://cge.cbs.dtu.dk/services/spatyper/)^45^.

### Phylogenetic tree construction

To prepare an input sequence for building a phylogenetic tree, SNVs present on Mobile Genetic Element (MGE) were discarded and only homozygous and biallelic SNVs were concatenated into the SNV input sequence (in-house script). Multiple sequence alignment of five ST764 isolates and N315, representing an outgroup, were tested for recombination signals by using Recombination Detection Program v4.10 (RDP4)^46^ that includes seven well-known recombination checking routines, namely RDP, GENECONV, Chimera, MaxChi, Bootscan, SiScan, and 3Seq. If more than four utilities reported recombination regions, these regions would be removed. These RDP-corrected sequences were used as the input for phylogenetic tree construction by the maximum likelihood (ML) method. PhyML version 3.0 was used to construct an ML-based phylogeny which can be visualized by the SeaView program^47,48^. That includes seven well-known recombination checking routines, namely RDP, GENECONV, Chimera, MaxChi, Bootscan, SiScan, and 3Seq. If more than four utilities reported recombination regions, these regions would be removed. These RDP-corrected sequences were used as the input for phylogenetic tree construction by the maximum likelihood (ML) method. PhyML version 3.0 was used to construct an ML-based phylogeny which can be visualized by the SeaView program^49^.

### ACME/SCC*mec* type II

The structure of ACME/SCC*mec* type II was determined by identification of *arcA*, *arcB*, *arcC*, *arc*D, *opp3* and *ccrC* using the sequence of MRSA USA300, (GenBank Accession: CP000255.1) as reference. Identification of pUB110 and cJR1 was done using N315 and NCTC10442 (SCC*mec* type I, GenBank Accession: UHCH01000003.1) as reference, respectively.

### Virulence gene identification

Genes present in three pathogenicity islands (SaPIn1, SaPIn2 and SaPIn3) were identified, which are SaPIn1: [*tst*, *sea, sec*, *sel, sek*], SaPIn2: [*set*6 to *set15*] and SaPIn3: [*seg*, *sei, sem*, *sen*, *seo*]. The presence of other enterotoxins (*seb* (AB630021.1), *sed*, *see*, *sep*, *seq*), exfoliative toxins (*eta*, *etb*), leukocidin (*lukE*-*lukD*), panton-valentine leukocidin (*pvl*), hemolysins (*hla*, *hld*, *hlb*, *hlgA*, *hlgB* and *hlgC*), and adhesin genes (*icaA*, *sdrC*-*E*, *ebpS*, *clfA*, *clfB*, *cna*, *bbp*, *femA*, *fnb*A and *fnb*B), were also verified. The *SA1320* gene was checked for the presence of an insertion in the middle of the genome by blasting the assembled contigs with *SA1320* sequence of N315.

The following sequences were used as reference: MRSA N315/SCC*mec* II/ST5/ACMEII (GenBank accession: BA000018.3)^50^, MRSA NN54/SCC*mec* II/ST764 (GenBank accession: BAFI01000000)^12^, MRSA PT2/SCC*mec* II/ST5/*seb* (GenBank accession: AB630021.1)^51^, MRSA H0 5096_0412/SCC*mec* IV/ST22 (GenBank accession: HE681097)^52^, JKD6008/SCC*mec* III/ST239 (GenBank accession: CP002120), and TW20/SCC*mec* III/ST239 (GenBank accession: FN433596)^53^.

The in-house script for converting SNV data in VCF file format to SNV concatenated sequences in fasta file format was deposited at https://github.com/voravich1/TB_platform/blob/5ef4ed3ddd93dd25df418a6fa632435663fc6f31/tb_convert_vcf_to_fasta_backbone.py.

## Supplementary Information

Supplementary Information.
