# Supplementary material for: Molecular characterization of methicillin-resistant Staphylococcus aureus genotype ST764-SCCmec type II in Thailand
Source: Sci Rep. 2022 Feb 8;12:2085. doi: 10.1038/s41598-022-05898-1 (PMC8826912; doi:10.1038/s41598-022-05898-1)
Supplement: Supplementary file 1 — Supplementary Information. [file 41598_2022_5898_MOESM1_ESM.docx]

**Supplementary Information for the manuscript**

**Molecular characterization of Methicillin-resistant *Staphylococcus aureus* Genotype ST764-SCC*mec* type II in Thailand**

Sumalee Kondo^1,^*, Pimonwan Phokhaphan^2^, Sissades Tongsima^2^, Chumpol Ngamphiw^2^, Worawich Phornsiricharoenphant^2^, Wuthiwat Ruangchai^3^, Areeya Disratthakit^4^, Pholawat Tingpej^1^, Surakameth Mahasirimongkol^4^, Aroonlug Lulitanond^5^, Anucha Apisarnthanarak^1^, Prasit Palittapongarnpim^3,^*

This supplementary PDF file includes the following information:

Supplementary Tables S1-S4

Supplementary Figure S1

| Strain code |  | SCC*mec* type | ST | *spa* type | **%** Reads uniquely mapped^†^ | WGS coverage^†^ | WGS average  depth^†^ | Number of  SNVs^†^ |
| --- | --- | --- | --- | --- | --- | --- | --- | --- |
| SATU130 |  | II | 764 | NA | 94% | 98% | 74X | 261 |
| SATU131 |  | II | 764 | t439 | 95% | 97% | 68X | 258 |
| SATU132 |  | II | 764 | t045 | 94% | 97% | 73X | 238 |
| SATU133 |  | III | 239 | t037 | 90% | 88% | 70X | 22720 |
| SATU134 |  | II | 764 | t439 | 95% | 97% | 85X | 258 |
| SATU135 |  | III | 22 | NA | 91% | 90% | 63X | 31919 |
| SATU136 |  | II | 764 | t045 | 94% | 96% | 77x | 237 |

ST, sequence type; NA, not available.

^†^Using N315 as the reference genome.

**Supplementary Table S1.** Genetic and genomic profiles of seven HA-MRSA isolates

| Sample | ST | Insertion | | | | | | | |
| --- | --- | --- | --- | --- | --- | --- | --- | --- | --- |
|  |  | **g.ASM964v1:8342_8343ins [unknown]** | **g.ASM964v1:676384_676385ins [unknown]** | **g.ASM964v1:941411_941412ins [unknown]** | **g.ASM964v1:1104595_1104596ins [unknown]** | **g.ASM964v1:1527689_1527690ins [unknown]** | **g.ASM964v1:477932_477933ins [unknown]** | **g.ASM964v1:1775670_1775671ins [unknown]** | **g.ASM964v1:2313237_2313238ins [unknown]** |
| SATU136 | 764 | + | - | + | + | + | - | - | - |
| SATU132 | 764 | + | - | + | + | + | - | - | - |
| SATU134 | 764 | + | + | + | + | + | - | - | - |
| SATU131 | 764 | + | + | + | + | + | - | - | - |
| SATU130 | 764 | + | + | + | + | + | - | - | - |
| SATU133 | 239 | - | - | - | - | - | + | + | + |
| SATU135 | 22 | - | - | - | - | - | + | + | + |

ST, sequence type; unknown, unknown length insertion element; +, found; -, not found.

**Supplementary Table S2.** Insertion events found in the MRSA isolates

| Strain code | ST | Deletion | | | | | | | | | | | | |
| --- | --- | --- | --- | --- | --- | --- | --- | --- | --- | --- | --- | --- | --- | --- |
|  |  | **g.ASM964v1:383663_383683del** | **g.ASM964v1:823521_823580del** | **g.ASM964v1:1608977_1609107del** | **g.ASM964v1:2068598_2068612del** | **g.ASM964v1:2793533_2793544del** | **g.ASM964v1:117451_119810del** | **g.ASM964v1:188291_188572del** | **g.ASM964v1:680355_680488del** | **g.ASM964v1:1021622_1021646del** | **g.ASM964v1:1398974_1399203del** | **g.ASM964v1:1917340_1917352del** | **g.ASM964v1:2094194_2094435del** | **g.ASM964v1:2536852_2537646del** |
| SATU136 | 764 | - | + | + | + | + | - | - | - | - | - | - | - | - |
| SATU132 | 764 | - | + | + | + | + | - | - | - | - | - | - | - | - |
| SATU134 | 764 | + | + | - | + | + | - | - | - | - | - | - | - | - |
| SATU131 | 764 | + | + | - | + | + | - | - | - | - | - | - | - | - |
| SATU130 | 764 | + | + | - | + | + | - | - | - | - | - | - | - | - |
| SATU133 | 239 | - | - | - | - | - | + | + | + | + | + | + | + | + |
| SATU135 | 22 | - | - | - | - | - | + | + | + | + | + | + | + | + |

ST, sequence type; +, found; -, not found.

**Supplementary Table S3.** Deletion events found in the MRSA isolates

| Primer | Oligonucleotide sequence **(**5'-3'**)** | Amplicon size **(**bp**)** | SCC*mec* type |
| --- | --- | --- | --- |
| Type I-F  Type I-R | GCT TTA AAG AGT GTC GTT ACA GG  GTT CTC TCA TAG TAT GAC GTC C | 613 | I |
| Type II-F  Type II-R | CGT TGA AGA TGA TGA AGC G  CGA AAT CAA TGG TTA ATG GAC C | 398 | II |
| Type III-F  Type III-R | CCA TAT TGT GTA CGA TGC G  CCT TAG TTG TCG TAA CAG ATC G | 280 | III |
| Type IVc-F  Type IVc-R | ACA ATA TTT GTA TTA TCG GAG AGC  TTG GTA TGA GGT ATT GCT GG | 200 | IVc |

**Supplementary Table S4.** Primers used for SCC*mec* typing


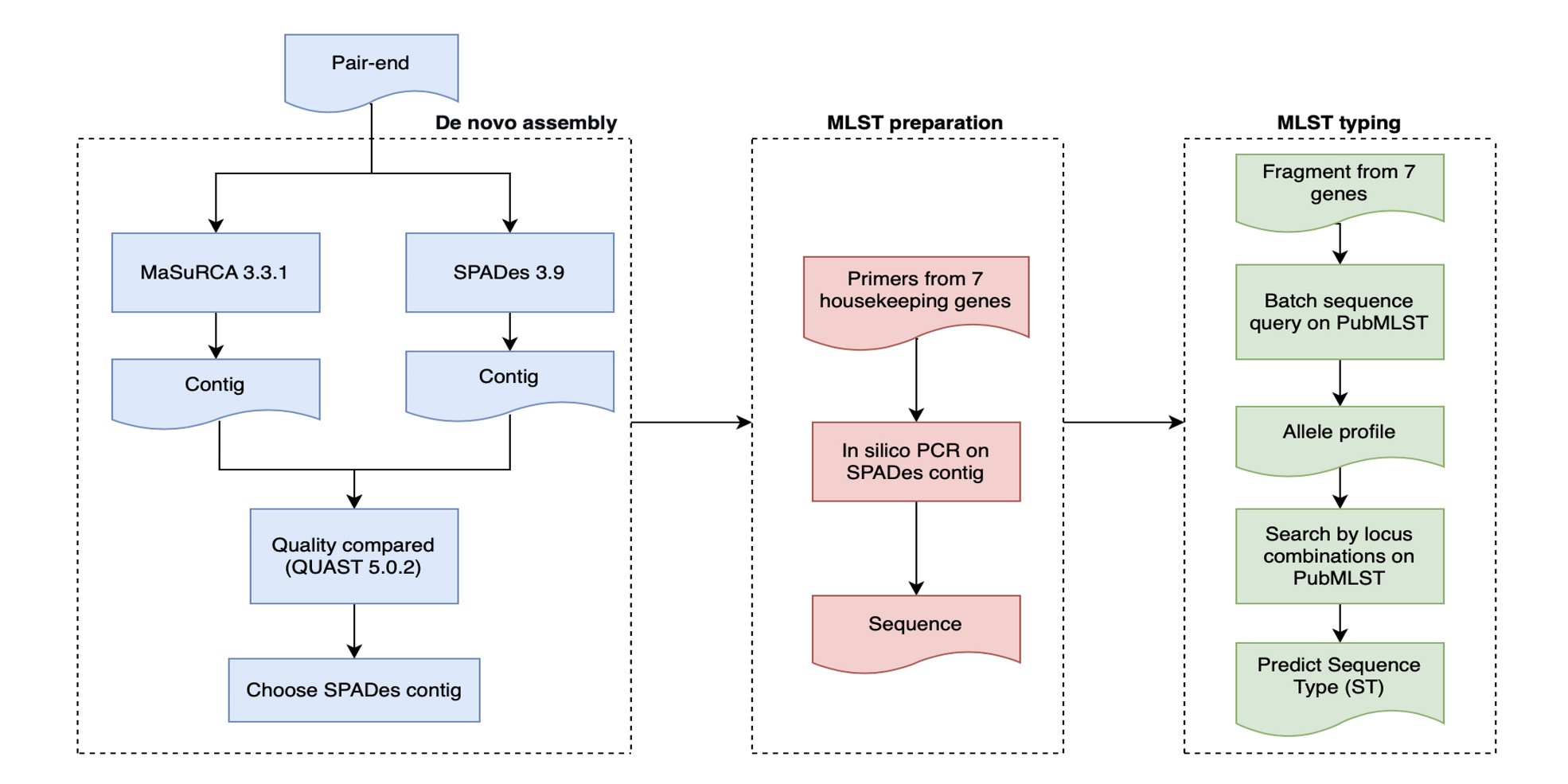


**Supplementary Figure S1.** De novo assembly and MLST workflow
